# The GluN3A subunit exerts a neuroprotective effect in brain ischemia and the hypoxia process

**DOI:** 10.1042/AN20130009

**Published:** 2013-08-29

**Authors:** Hui Wang, Haitao Yan, Shuzhuo Zhang, Xiaoli Wei, Jianquan Zheng, Jin Li

**Affiliations:** Beijing Institute of Pharmacology and Toxicology, 27 Taiping Road, Beijing 100850, People's Republic of China

**Keywords:** brain hypoxia and ischemia, excitotoxicity, GluN3A, *N*-methyl-D-aspartate receptor (NMDAR), oxygen and glucose deprivation (OGD), 2VO, two-vessel occlusion, CNS, central nervous system, DAPI, 4′,6-diamidino-2-phenylindole, FCM, flow cytometry, gDNA, genomic DNA, HBSS, Hanks’ balanced salt solution, MTT, 3-(4,5-dimethylthiazol-2-yl)-2,5-diphenyl-2*H*-tetrazolium bromide, NMDAR, *N*-methyl-D-aspartate receptor, NO, nitric oxide, OGD, oxygen and glucose deprivation, PI, propidium iodide, RT, reverse transcription, S–D, Sprague–Dawley, TBST, TBS containing 0.1% Tween-20, TTC, triphenyltetrazolium chloride

## Abstract

NMDARs (*N*-methyl-D-aspartate receptors) mediate the predominantly excitatory neurotransmission in the CNS (central nervous system). Excessive release of glutamate and overactivation of NMDARs during brain ischemia and the hypoxia process are causally linked to excitotoxicity and neuronal damage. GluN3 subunits, the third member of the NMDAR family with two isoforms, GluN3A and GluN3B, have been confirmed to display an inhibitory effect on NMDAR activity. However, the effect of GluN3 subunits in brain ischemia and hypoxia is not clearly understood. In the present study, the influence of ischemia and hypoxia on GluN3 subunit expression was observed by using the 2VO (two-vessel occlusion) rat brain ischemia model and cell OGD (oxygen and glucose deprivation) hypoxia model. It was found that GluN3A protein expression in rat hippocampus and the prefrontal cortex was increased quickly after brain ischemia and remained at a high level for at least 24 h. However, the expression of the GluN3B subunit was not remarkably changed in both the animal and cell models. After OGD exposure, rat hippocampal neurons with GluN3A subunit overexpression displayed more viability than the wild-type neurons. NG108-15 cells overexpressing GluN3A presented pronounced resistance to glutamate insult. Blocking the increase of intracellular Ca^2+^ concentration may underlie the neuroprotective mechanism of up-regulated GluN3A subunit. Suppressing the generation of hydroxyl radicals and NO (nitric oxide) is probably also involved in the neuroprotection.

## INTRODUCTION

NMDARs (*N*-methyl-D-aspartate receptors) are ionotropic glutamate receptors that mediate the vast majority of excitatory neurotransmissions in the mammalian CNS (central nervous system) and play critical roles in neurodevelopmental, neurophysiological and neuropathological processes (Traynelis et al., 2010). Conventional NMDARs are believed to form a heterotetramer between two GluN1 and GluN2 subunits and to require co-activation by glutamate and glycine. The third member of the NMDA (*N*-methyl-D-aspartate) family, the GluN3 subunit, was discovered in 1995 (Ciabarra et al., 1995; Sucher et al., 1995; Forcina et al., 1995). New evidence shows that functional NMDARs are comprised of GluN1 and at least one GluN2 subunit or of GluN1 and both GluN2 and GluN3 subunits. The GluN3 subunit has been proven to display an inhibitory effect on NMDAR activity (Henson et al., 2010; Low and Wee, 2010). Compared with NMDAR formed by GluN1/GluN2 subunits, coexpression of GluN3 with GluN1 and GluN2 subunits decreases the amplitude of NMDAR current, unitary conductance, Ca^2+^ permeability and Mg^2+^ sensitivity (Sucher et al., 1995; Das et al., 1998; Nishi et al., 2001; Chatterton et al., 2002; Matsuda et al., 2002; Sasaki et al., 2002; Pina-Crespo et al., 2010). The GluN3 subunit has two isoforms: GluN3A and GluN3B. Both show restricted spatiotemporal distributions, with GluN3A being predominantly expressed during early development, although the expression in certain neuronal populations persists in adults (Wong et al., 2002). GluN3B was once thought to be expressed only in motoneurons (Nishi et al., 2001). However, it has been found to be expressed in the rat hippocampus, cerebral cortex, caudoputamen, nucleus accumbens, cerebellum and lumbar sections of the spinal cord. This distribution is the same as that of GluN1 (Wee et al., 2008).

Ischemic cerebrovascular disease is of great concern because of its high morbidity, mortality and pronounced tendency to cause disability (Woodruff et al., 2011). The pathophysiology of brain ischemia is complicated and involves numerous processes, including increased intracellular Ca^2+^ levels and excitotoxicity. During brain ischemia, glutamate is released by reversed operation of glutamate uptake carriers. The extracellular accumulation of glutamate activates NMDARs and leads to an excessive Ca^2+^ influx through NMDAR-operated channels, which disrupts neuronal intracellular homeostasis and consequently triggers excitotoxicity (Lipton and Rosenberg, 1994; Albrecht et al., 2010). The GluN3 subunits can decrease excitotoxicity by depressing NMDAR activity. Our previous study showed that the expression of GluN3A on rat hippocampus had a time-dependent pattern. Resistance to ischemic injury was positively related to the increased expression level of GluN3A in rat hippocampus (Wang et al., 2012). Transgenic and knockout mice also indicate the neuroprotective role of GluN3A (Nakanishi et al., 2009). However, the effects and mechanism of GluN3 subunits that may perform during brain ischemia and hypoxia remain unclear. In the present study, the influence of brain hypoxia and ischemia on GluN3 expression was observed by setting up a rat 2VO (two-vessel occlusion) brain ischemia and hypoxia model and cell OGD (oxygen and glucose deprivation) model. The effects of GluN3A subunits on brain hypoxia and ischemia were observed using a GluN3A overexpression cell model. The results revealed that the GluN3A subunit was up-regulated during ischemia and hypoxia and exerted a neuroprotective effect against ischemia- and hypoxia-induced neuronal damage. Blocking the rise of intracellular Ca^2+^ levels may underlie the neuroprotective mechanism of GluN3A subunits, and suppression of hydroxyl radicals and NO (nitric oxide) generation may be also involved in the protection.

## MATEREIALS AND METHODS

### Animals

Male S–D (Sprague–Dawley) rats weighing 180–220 g were maintained at a constant temperature (22±1°C) and relative humidity (50%) under a regular light–dark cycle (lights on at 7 a.m. and off at 7 p.m.) with free access to food and water. The animals were treated in accordance with the NIH Guidelines for the Care and Use of Laboratory Animals (1996) and with those of the local ethical committee. All efforts were made to minimize animal suffering and to reduce the number of animals required.

### Cells

#### Isolation of primary neurons from rat brains and subsequent cell culture

Hippocampal and cortical neurons were isolated using a combination of established methods (Kay and Wong 1986; Das et al., 1998). Briefly, rats born within 12 h were decapitated and the brains were promptly removed and placed in a storage vessel with cooled HBSS (Hanks’ balanced salt solution, 137 mM NaCl, 1 mM NaHCO_3_, 0.35 mM Na_2_HPO_4_, 5.4 mM KCl, 0.45 mM KH_2_PO_4_, 1.25 mM CaCl_2_, 0.5 mM MgSO_4_, 0.5 mM MgCl_2_, 5 mM HEPES and 20 mM glucose, pH 7.4). Tissues of the hippocampus and the prefrontal cortex were segregated individually and enzymatically dissociated by 0.25% Trypsin (Sigma). They were then mechanically dispersed into a single-cell suspension and plated onto a 35 mm dish coated with 0.1 mg/ml poly-L-lysine (Sigma) at high density (5×10^5^ cells per 35 mm dish). Cells were maintained in neurobasal medium supplemented with B27, 0.5 mM glutamine in the incubator (37°C, 8% CO_2_) for 1–3 weeks and were used for Western blot or transfection.

#### GluN3A overexpression

The GluN3A-pcDNA3.1^(+)^ plasmid, which had a copy of the *G418* resistance gene, was kindly donated by Dongxian Zhang from the Sanford-Burnham Medical Research Institute. As per the manufacturer's recommended protocol, 5 μg of GluN3A-pcDNA3.1^(+)^ cDNA was transfected into NG108-15 cells (5×10^6^, about 85% confluence) in a 100 mm dish with10 μl of Lipofectamine™ 2000 (Invitrogen). Clones with resistance to G418 (800 μg/ml) were selected and expanded. The expression of GluN3A protein from these clones was assessed using Western blot analysis. Stable transfection was achieved in NG108-15 cells. Wild-type and transfected NG108-15 cells were grown in tissue culture as previously described (Adie and Milligan, 1994), except that the transfected cells were also maintained in the presence of G418 (800 μg/ml). Control cells were transfected with the pcDNA3.1^(+)^ plasmid without the *GluN3A* gene.

cDNA of GluN3A-pcDNA3.1^(+)^ (2 μg) was transfected into hippocampal neurons cultured in a 35 mm dish by using 4 μl of Lipofectamine™ 2000, as recommended, on the sixth cultured day. Neurons were maintained in an incubator (37°C, 5% CO_2_) for 42–48h followed by cell injury models. The estimated transfection efficiency was about 15%, based on the results of parallel control experiments by transfecting GFP (green fluorescent protein)-containing plasmids into neurons.

### Rat brain ischemia and hypoxia models and cell injury models

#### 2VO in the rat brain ischemia and hypoxia model

**Figure 1:**
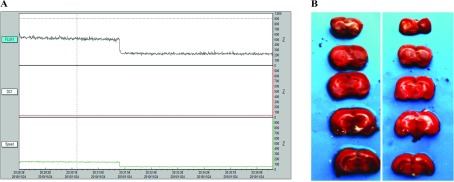
The 2VO rat brain ischemia model was verified using laser Doppler flowmetry and TTC staining (**A**) Animals with blood flow (FLUX1) reduced at least 50% were used as 2VO models. ‘FLUX1, DC1 and Speed’ indicated blood flux, intensity of all detected light and the mean speed of blood, respectively. (**B**) After 3 h of occlusion, only a small part (white part) of the brain around the hippocampus was injured (left). Nearly the whole hippocampus was infarcted after 24 h of occlusion (right).

Male S–D rats weighing 180–220 g (Animal Center, AMMS) were anesthetized with 4% (w/v) chloral hydrate (5.5 mg/10 g, intraperitoneal injection) and surgically prepared for use in the brain ischemia models according to previously described methods (Cechetti et al., 2010). During surgery, room temperature was maintained within 20–25°C, and two surgical lights were used for illumination and maintenance of the animals’ normal temperature. Each surgery was completed within 5 min. A sham operation was performed on all control animals. Occlusion was verified by using laser Doppler flowmetry (Moor) to monitor relative cerebral blood. As shown in Figure 1(A), animals in which blood flow was reduced by at least 50% were used as 2VO models. TTC (2,3,5-triphenyltetrazolium chloride) staining was also performed to determine the severity of brain injury. Infarcted tissue could not be stained with TTC and was white after TTC staining. As shown in Figure 1(B), only a small part of the brain around the hippocampus was injured (left) after 3 h of occlusion. Nearly the whole hippocampus was infarcted after 24 h of occlusion (right). The animals were killed after brain ischemia lasting different periods of time and the brain tissues were frozen for storage in liquid nitrogen.

#### Cell OGD model

Rat hippocampal and prefrontal cortex neurons were cultured for 25 days and treated under OGD exposure conditions modified according to a previously described method (Zhang et al., 2011) with 300 mM sodium dithionite in the glucose-free Earle's medium (122 mM NaCl, 5.4 mM KCl, 2.6 mM NaH_2_PO_4_, 0.8 mM MgSO_4_, 1.8 mM CaCl_2_, 26.2 mM NaHCO_3_ and 20.1 mM HEPES) for 4 h. The wild-type cells and OGD cells were collected after OGD exposure and used for Western blot analysis.

#### Glutamate injury in NG108 cells

NG108-15 cells were exposed to 100 μM glutamate (Sigma) with 10 μM glycine (Sigma) for 30 min in an incubator (37°C, 5% CO_2_) to induce excitotoxicity as described previously (Gonzalez et al., 1998). Exposure was terminated by replacement with growth media. Cell death assessments were performed later. For groups treated with MK-801, a selective and non-competitive NMDAR antagonist (Wong et al., 1986), 100 μM MK-801, was added 30 min before glutamate injury.

### Analysis of NMDAR subunit protein

#### Brain tissue protein and cell protein preparation

In all experiments, for protein preparation, 100 μl of cold RIPA lysis buffer [50 mM Tris-acetate, 150 mM NaCl, 1% (v/v) NP-40, 0.1% (w/v) SDS, pH 7.4] and freshly mixed protease inhibitor cocktail (1 mM PMSF, 20 μg/ml benzamidine, 10 μM leupeptin and 1 μM pepstatin) was added to 10 mg of dissected brain tissue or 1×10^6^ cells. The brain tissue in the lysis buffer was homogenized in a Potter homogenizer (cells were not needed for this step). After lysis in ice for 20 min, tubes containing the tissue homogenates or cells were centrifuged at 12000 ***g*** for 15 min at 4°C. The supernatants were moved to new tubes and subsequently frozen for storage at −80°C. The protein concentrations were determined by using a BCA (bicinchoninic acid) protein assay (Thermo Scientific), and 50–100 μg of protein was loaded for SDS gel electrophoresis.

#### Western blot hybridization

In brief, SDS/PAGE was used to separate proteins that were then transferred to PVDF membranes. Membranes were blocked in TBST [tris-buffered saline containing 0.1% (v/v) Tween-20] and 5% (w/v) non-fat dried skimmed milk powder for 1 h at room temperature and incubated with primary antibodies for GluN1 (1:4000, Abcam, ab17345), GluN2B (1:1000, Millipore, Cat. #06-600), GluN3A (1:2000, Upstate Biochemicals, Cat. #07-356), GluN3B (1:2000, Upstate Biochemicals, Cat. #07-351) and β-actin (1:10000) overnight at 4°C, respectively. Before incubation of an HRP (horseradish peroxidase)-conjugated secondary antibody with membranes at room temperature for 1 h, membranes were washed three times (10 min each) with TBST to remove unbound primary antibodies. Then membranes were washed three times (10 min each) with TBST and bands were identified by use of the ECL (enhanced chemiluminescence) Plus Western blotting detection system (Kodak). Images were scanned and quantified using ImageJ software (version 1.33 u; National Institutes of Health, Bethesda, MD, U.S.A.).

### Analysis of RNA of GluN3A

#### RNA extraction

Total RNA was extracted using Trizol Reagent (Invitrogen) according to the instructions of the manufacturer. The yield of total RNA was determined by measuring the absorbance (260–280 nm) of ethanol-precipitated aliquots of the samples by using spectrophotometer (Pharmacia). Isolated RNA was finally frozen at −80°C until further processing.

#### RT (reverse transcription)

A PrimeScript® RT reagent Kit with gDNA (genomic DNA) Eraser (TaKaRa) was used. gDNA in samples was eliminated first. The reaction mixture (10 μl) contained 1 μg of total RNA, 1 μl of gDNA Eraser, 2 μl of 5×Buffer and RNase-free H_2_O. Reactions were performed for 2 min at 42°C. Then the purified RNA samples were reverse transcribed. Each mixture (20 μl) contained 1 μg of total RNA, 4 μl of 5×RT buffer, 1 μl of PrimeScript RT Enzyme Mix, 1 μl of RT Primer Mix and RNase-free H_2_O. Reactions were performed for 15 min at 37°C, and terminated by 5 s at 85°C. The reaction mixture was maintained at −20°C until it was used for PCR amplification.

#### qRT-PCR (quantitative real-time PCR)

qRT-PCR of GluN3A mRNA was performed in triplicate using gene-specific primers and SYBR Green (SYBR Premix Ex TaqTM, TaKaRa). Oligonucleotide primers were designed by using Primer Express 3.0 software (Applied Biosystems). The primer sequences of GluN3A were P1:5′-CTTCCATGCTGGATCGTCTGT-3′ and P2:5′-TCTTGAACTTGATTGGCACTGTGT-3′. As an internal control for normalization, PCRs were performed concurrently with the amplification of a reference gene, β-actin with the primers P3:5′-GGGAAATCGTGCGTGACATT-3′ and P4:5′-GCGGCAGTGGCCATCTC-3′. Real-time PCR was performed on CFX96 Touch™ Real-Time PCR Detection System (Bio-Rad) with the following thermal cycler settings: 40 cycles of 30 s at 95°C and 30 s at 63°C. The reaction mixture (25 μl) consisted of 0.5 μl of cDNA (1 μg), 5 nM of each primer, 12.5 μl of SYBR Ex Taq and 11 μl of RNase-free H_2_O. The relative change in the mRNA expression studied after ischemia was determined using Bio-Rad Manager TM Software.

### Analysis of cell resistance to injury with overexpressing GluN3A

#### PI/DAPI double stain

DAPI (Sigma) is a blue fluorescent dye that stains the condensed chromatin in apoptotic cells more brightly than normal chromatin. PI (Sigma), a red fluorescent dye, only permeates dead cells. The double stain provides a rapid and convenient way of assaying normal, apoptotic and dead cells. After OGD exposure, double staining was performed directly on living cells without fixation, as follows: DAPI and PI were added to the culture medium at a final concentration of 1 μg/ml for each reagent and incubated for 10 min at 37°C in the dark. Medium containing floating cells was gently removed and cells were observed using fluorescence microscopy (Olympus). Three separate experiments were performed. For each experiment, and each incubation time, at least three microscopic fields were photographed.

#### MTT assay

The MTT assay is commonly used to assess cell viability (Hengartner, 2000). After cell injury, 10 μl of MTT (Amresco) suspended in PBS (5 mg/ml) was added to each well and incubated for 4 h at 37°C in the dark. MTT solution was then aspirated and formazan was instantly dissolved by the addition of 150 μl of DMSO. The absorption was read with the multi-label counter (Tecan). Three separate experiments were performed. For each experiment, each data point was assessed in at least five replications.

#### FCM (flow cytometry) assay

An Annexin V-FITC apoptosis kit (Saibao) was used. After digestion with 0.25% trypsin, cells were gathered and rinsed with ice-cold PBS and then resuspended in 200 μl of binding buffer according to the manufacturer's instructions. Then 10 μl of Annexin V stock solutions were added to the cells and incubated for 15 min at room temperature in the dark. After 15 min incubation, 300 μl of binding buffer with 5 μl of PI was added to the cell mixture. The mixture was immediately analyzed by using FCM (BD).

### Analysis of anti-injury mechanisms

#### Ca^2+^ imaging assay

Cells were loaded with the fluorescent Ca^2+^indicator fluo4-AM (Invitrogen). According to the manufacturer's instructions, the medium was replaced with HBSS containing 2 μM fluo4-AM and returned to the incubator (37°C, 5% CO_2_). After 30 min of incubation, cells were rinsed three times with HBSS and placed on the stage of a laser scanning confocal microscope (PerkinElmer). Images of cells were obtained and analyzed using the Velocity imaging system (version 6.0.1). Before glutamate injury, images were obtained for several minutes to establish a stable baseline Ca^2+^ measurement. Then 100 μM glutamate and 10 μM glycine were applied, and images were obtained at 2 s intervals.

#### NO measurement and hydroxyl radical measurement

A NO assay kit (Jiancheng) and hydroxyl radical assay kit (Jiancheng) were used independently to monitor the NO and hydroxyl radicals in cells after glutamate injury. Procedures were performed according to the manufacturer's instructions. Three separate experiments were performed. For each experiment, each data point was replicated at least three times.

### Data analysis

Data were expressed as the means±S.E.M. Pairwise comparisons were made by Student's *t* test. The means of three or more groups were compared with one-way ANOVA and Dunnett's test. The statistical comparisons were considered significant at probability level <0.05.

## RESULTS

### Expression of NMDAR subunits in hippocampal and prefrontal cortex neurons in the rat 2VO brain ischemia and hypoxia model

**Figure 2:**
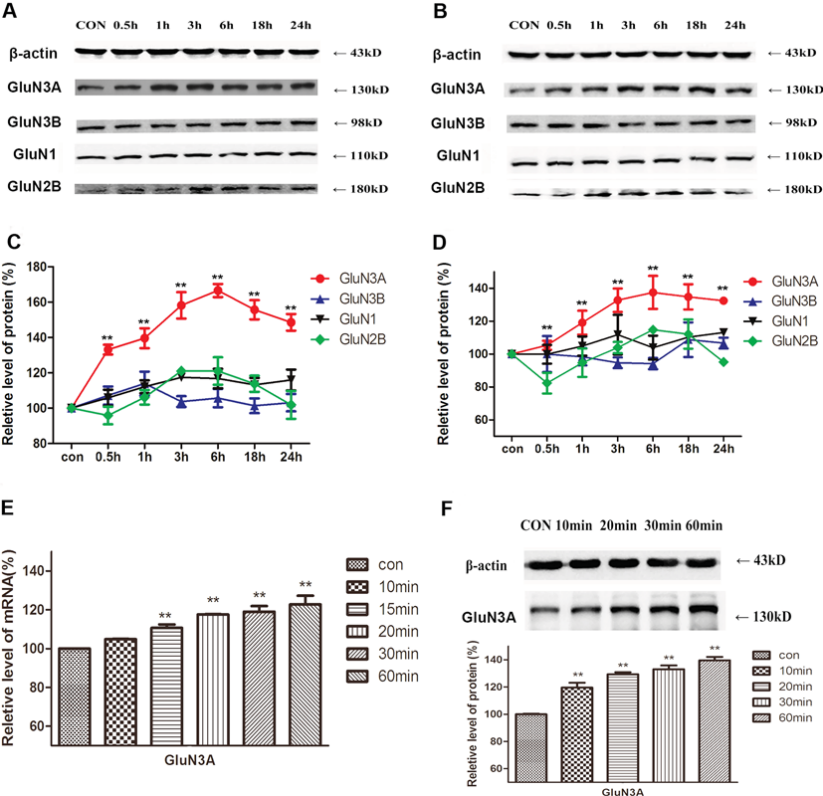
The effects of brain ischemia and hypoxia on the expression of NMDAR subunits in the hippocampus and prefrontal cortex (**A**, **B**) The protein levels of different NMDAR subunits were detected by Western blot analysis in hippocampus (**A**) and prefrontal cortex (**B**). (**C**, **D**) Quantitative analysis showed significant changes of NMDAR subunits in hippocampus (**C**) and prefrontal cortex (**D**). Protein of GluN3A increased significantly after the brain ischemia process in both tissues. GluN3A mRNA levels (**E**) and protein levels (**F**) both increased after 10 min of occlusion of the 2VO model. Quantitative data are presented as means±S.E.M. (*n*=5/group). **P<0.01 compared with control, one-way ANOVA, Dunnett's multiple comparison test.

The effects of brain ischemia and hypoxia on the expression of NMDAR subunits in the hippocampus and prefrontal cortex were observed in a rat 2VO model. The protein levels of different NMDAR subunits were detected by Western blot analysis and one set of experiment data is presented in Figures 2(A) and 2(B) for the hippocampus and prefrontal cortex, respectively. The quantitative analysis (Figures 2C and 2D) revealed that, among all the NMDAR subunits, GluN3A displayed a remarkable higher level of protein expression. Compared with the control group, the level of GluN3A protein was increased to 133±3, 140±6, 158±7, 167±4, 156±5 and 149±5% (*F*=106.4, *P*<0.01, *n*=5) in the hippocampus, and to 105±3, 119±7, 133±7, 137±10, 135±7 and 132±1% in the prefrontal cortex (*F*=29.80, *P*<0.01, *n*=5) after 0.5, 1, 3, 6, 18 and 24 h of ischemia, respectively. Ischemia-induced changes in the expression of the subunits of GluN3B, GluN1 and GluN2B were similar with those observed in the hippocampus, of which changes of GluN3B protein levels were not significant, GluN1 expression showed little increase, whereas GluN2B decreased at first, increased after 3 h, peaked at 6 h, and decreased to original levels at 24 h after ischemia.

Real-time PCR and Western blot revealed a quick increase in GluN3A mRNA (*F*=30.37, *P*<0.01, *n*=5, Figure 2E) and protein levels (*F*=94.40, *P*<0.01, *n*=5, Figure 2F) in the hippocampus after only 10 min of brain ischemia.

### Expression of NMDAR subunits in hippocampal and prefrontal cortex neurons in the OGD model

**Figure 3:**
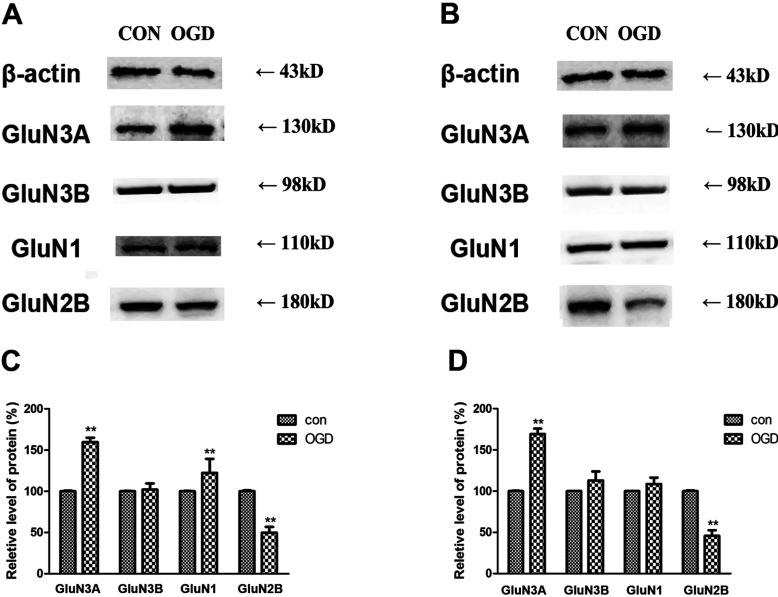
Changes in the expression of NMDAR subunits after OGD exposure (**A**, **B**) Western blot analysis showed levels of expression NMDAR subunits in hippocampal neurons (**A**) to be similar to the level of expression in the neurons of the prefrontal cortex (**B**). (**C**, **D**) Quantitative analysis showed significant changes in the concentrations of NMDAR subunits in hippocampal (**C**) and prefrontal cortex neurons (**D**). Data are presented as means±S.E.M. (*n=*3/group).**P<0.01 relative to the control, *t* test.

OGD is a cell model that simulates ischemia and hypoxia *in vitro*. Here, we exposed two different kinds of neurons, hippocampal (Figure 3A) and prefrontal cortex neurons (Figure 3B) to OGD and investigated changes in the level of protein expression of NMDAR subunits by using Western blot analysis. It was found that GluN3A increased after exposure to OGD by 160±5 and 170±4% (*P*<0.01, *n*=3) in hippocampal and prefrontal cortex neurons, respectively, relative to the control group. No significant changes in GluN3B were observed in either type of neuron. Protein levels of GluN1 increased by 122±7% (*P*<0.01, *n*=3) and 109±1%, respectively, relative to controls. GluN2B decreased by about 50% (*P*<0.01, *n*=3) in these two kinds of cells.

### Resistance to injury of cells overexpressing GluN3A

**Figure 4:**
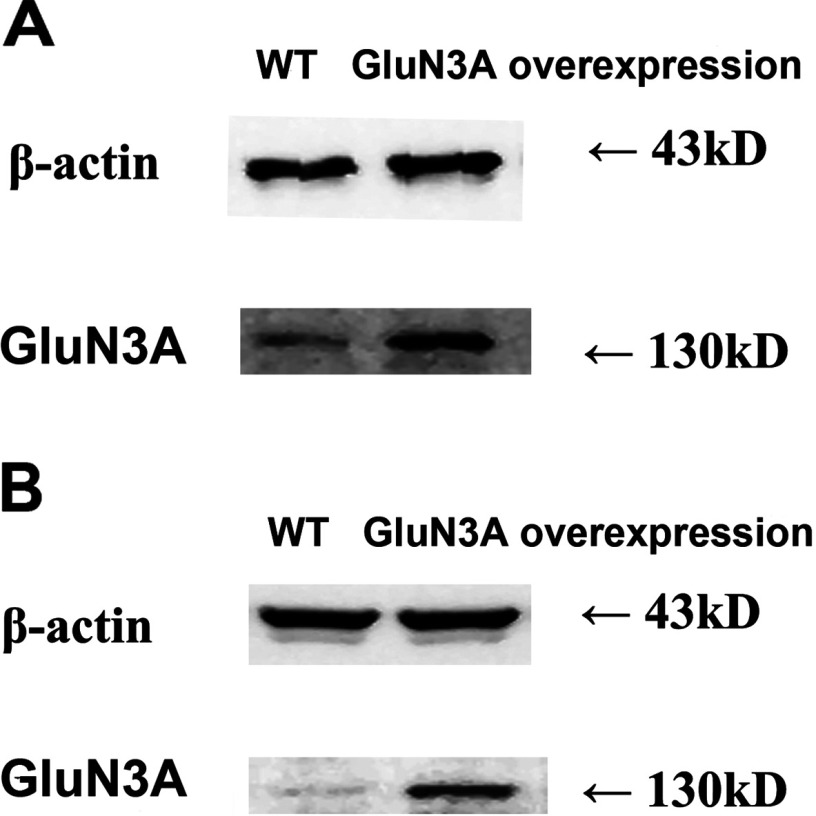
Overexpression of GluN3A subunit in hippocampal neurons (A) and NG108-15 cells (B) was verified by Western blot analysis

To observe the protective effect of the GluN3A subunit against ischemia and hypoxia, cells with GluN3A overexpression were achieved by transfection of GluN3A-pcDNA3.1^(+)^ plasmid into rat hippocampal neurons and NG108-15 cells, respectively. Western blot showed that GluN3A overexpression was achieved on rat hippocampal neurons (Figure 4A) and NG108-15 cells (Figure 4B), with the overexpressed efficiency of 25 and 80%, respectively.

**Figure 5:**
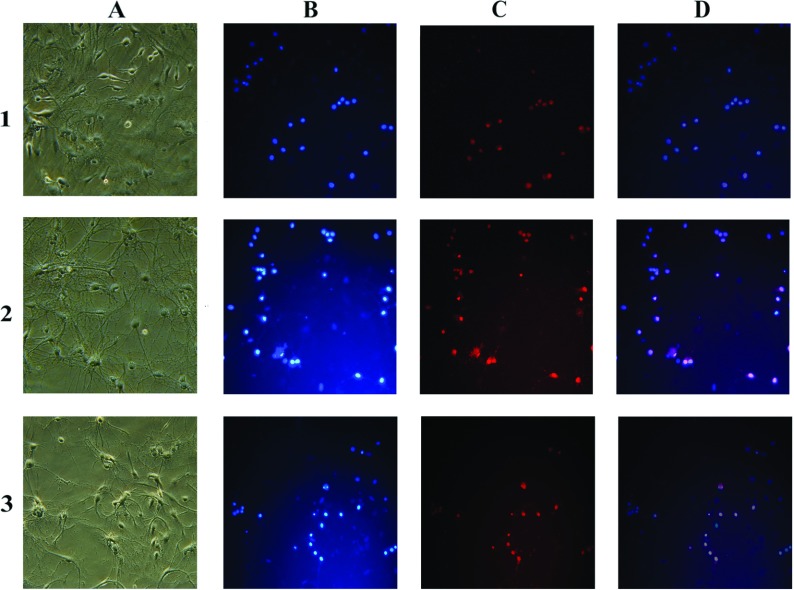
Double staining of hippocampal neurons with DAPI (D) and PI with or without OGD exposure The row number of 1, 2, and 3 represented control cells without OGD, control neurons with OGD, and GluN3A overexpressed neurons with OGD exposure, respectively. The capital letters for each column indicated the morphological pictures of neurons (**A**), neurons with DAPI stained (**B**), PI stained (**C**) and merging (**D**) of two images, respectively. Neurons with DAPI and PI staining distinguished the typical features of apoptosis (fragmented bright D^+^/PI^−^), necrosis (D^−^/PI^+^ or weak D^+^/PI^+^), and normal (D^+^/PI^−^). Merging (**D**) of the two images of (**B**) and (**C**) allowed detection of double-stained cells and indication of late apoptosis and dead neurons. The results suggested that hippocampal neurons overexpressing GluN3A subunits were more resistant to OGD exposure induced injury than wild-type cells.

The cell OGD model and glutamate injury model were used to compare the cell viability with and without GluN3A transfection. Hippocampal neurons overexpressing GluN3A subunits were more resistant to injury than wild-type cells as indicated by PI/DAPI double-stain results after OGD exposure (Figure 5).

**Figure 6:**
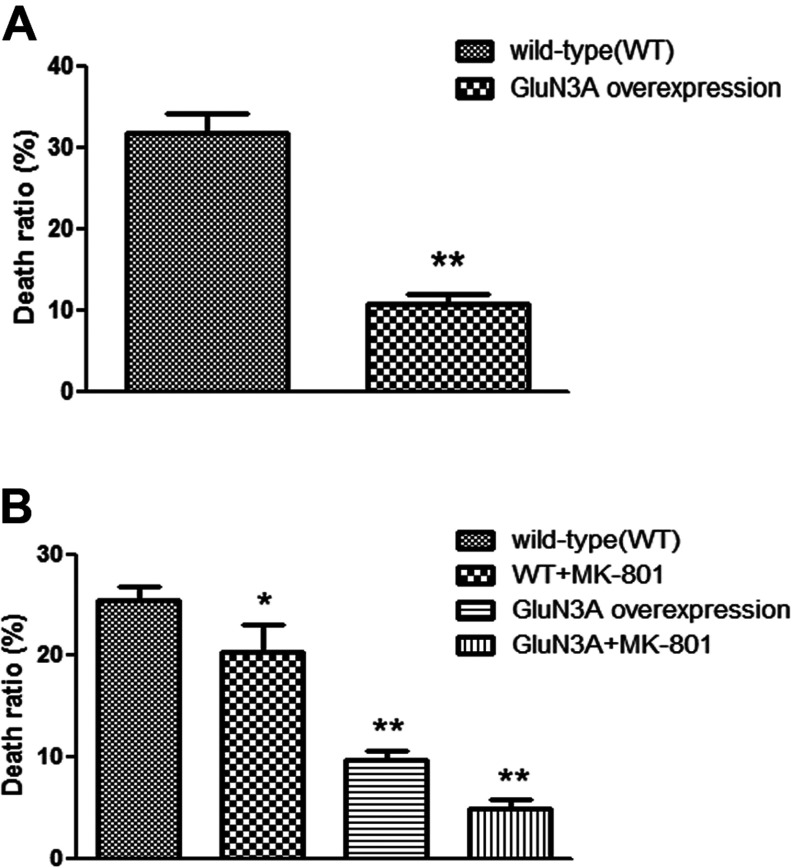
Relative ratio of cell death of hippocampal neurons (A) after OGD injury and NG108-15 cells (B) after glutamate injury was determined by MTT assay The sample absorbance was converted to relative death ratio. Data are presented as means±S.E.M., **P<0.01, *P<0.05 compared with vector alone (control).

MTT assay revealed that, among wild-type hippocampal neurons, OGD insult killed 31.7±2.5% of the cells. However, among the cells overexpressing GluN3A, the cell death rate attributable to OGD insult was only 10.7±1.2% (Figure 6A).

As shown in Figure 6(B), MTT assay showed that NG108-15 cells overexpressing GluN3A were more resistant to glutamate injury than wild-type cells. In NG108-15 cells without overexpressing GluN3A, glutamate insult resulted in a 25.4±1.5% death rate. The death rate dropped to 15.0±1.7% when the cells were pretreated with non-competitive NMDAR antagonist MK-801. Among NG108-15 cells overexpressing GluN3A, glutamate insult caused a death rate of only 9.6±0.9%. And the death rate was further lowered to 4.8±0.7% when MK-801 was also applied. The death rates of the three test groups were all significantly different from the control group (Figure 6B, *F*=97.16, *P*<0.01, *n*=3).

**Figure 7:**
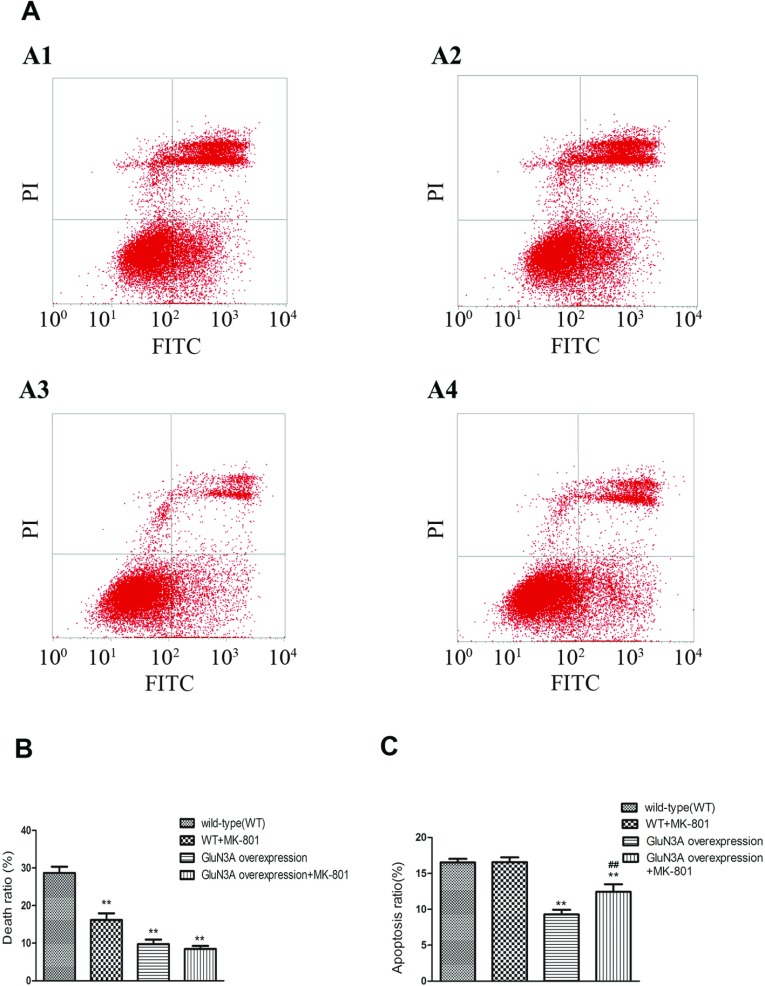
FCM results of NG108-15 cell viability after glutamate injury (**A**) FCM results. The lower left quadrants of each dot blot showed the viable cells (FITC^−^/PI^−^). The lower right quadrants represented the apoptotic cells (FITC^+/^PI^−^) and the upper right quadrants indicated the necrotic cell population (FITC^+^/PI^+^). Markers A1–A4 represented the WT (wild-type), WT+MK-801, GluN3A overexpressing and GluN3A+MK-801 cells, respectively. (**B**, **C**) Statistical analysis of the death ratio (**B**) and apoptosis ratio (**C**). Data were presented as means±S.E.M., **P<0.01, relative to vector alone (control), ^##^*P*<0.01, relative to the group with GluN3A overexpression and without MK-801 pretreatment.

FCM also indicated a lower death rate among GluN3A transfected cells subjected to glutamate insult (Figure 7). Cell death attributable to glutamate insult in wild-type NG108-15 cells, wild-type NG108-15 cells pretreated with MK-801, NG108-15 cells overexpressing GluN3A, and NG108-15 cells overexpressing GluN3A pretreated with MK-801 were 28.7±1.7, 16.2±1.8, 9.7±1.2 and 8.5±0.8%, respectively. The death rates among the three test groups were all significantly lower than that of the control group (Figure 7B, *F*=127.4, *P*<0.01, *n*=3). The group overexpressing GluN3A with MK-801 pretreatment showed the highest viability. However, the changes of apoptosis ratio displayed a different characteristic (Figure 7C). Only NG108-15 cells overexpressing GluN3A presented an apoptosis ratio (9.3±0.7%) lower than that of control group (16.5±0.5%, *P*<0.01, *n*=3). MK-801 had no effect on the apoptosis induced by glutamate insult in NG108-15 cells without overexpressing GluN3A with the ratio of 16.6±0.6%, and even increased the apoptosis ratio of NG108-15 cells overexpressing GluN3A from 9.3±0.7 to 12.4±1.0% (*P*<0.01).

### Generation of hydroxyl radicals and NO in NG108-15 cells overexpressing GluN3A after glutamate injury

**Figure 8:**
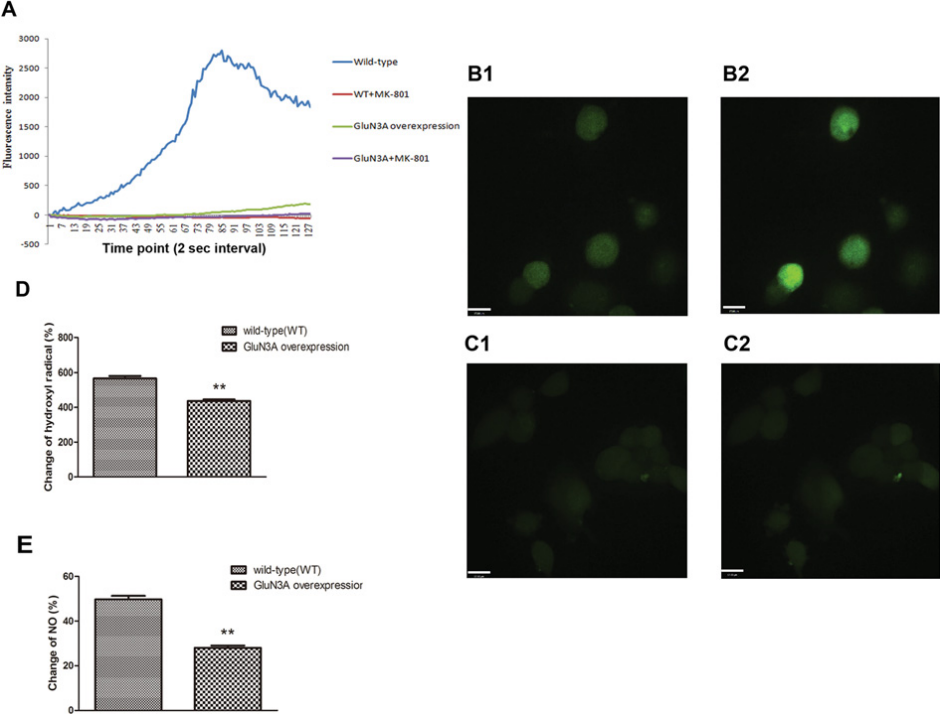
Possible mechanisms involved in the protective ability of GluN3A (**A**) Intracellular Ca^2+^ ion changes in NG108-15 cells overexpressing or not overexpressing GluN3A subunits after glutamate injury. Data were record at 2 s intervals for 4 min while glutamate and glycine were added. (**B**) After glutamate injury, a large amount of Ca^2+^ flowed into cells (**B2**, bright green) compared with the original status (**B1**, dark green) in WT (wild-type) cells. (**C**) Little change of intracellular Ca^2+^ signal in GluN3A overexpressing cells before (**C1**) and after (**C2**) glutamate injury. (**D**, **E**) Changes of hydroxyl radical and NO after glutamate injury. NG108-15 cells with or without GluN3A overexpression were monitored, and sample absorbance was converted to a change ratio. Data were presented as means±S.E.M., **P<0.01 relative to vector alone (control).

Fluo4-AM served as a Ca^2+^ indicator and was used to monitor changes in intracellular Ca^2+^ levels after glutamate insult. Wild-type cells turned bright green quickly, indicating influx of large amounts of Ca^2+^ into cells (Figures 8A and 8B). However no such obvious changes occurred in cells overexpressing GluN3A or cells pretreated with MK-801. This suggested that cells overexpressing GluN3A were less permeable to Ca^2+^. Figures 8(D) and 8(E) show changes in the concentrations of hydroxyl radicals and NO after glutamate injury in each group. Compared with wild-type cells, the concentrations of hydroxyl radicals and NO level in cells overexpressing GluN3A decreased from 567±14 and 49.7±1.6% to 437±8.6 and 28.0±1% after glutamate insult, respectively (*P*<0.05, *n*=3).

## DISCUSSION

The GluN3 subunits (GluN3A and GluN3B) are newly recognized players in the well-established field of NMDARs. GluN3A protein has been shown to be widely expressed in the postnatal and adult mammalian CNS (Wong et al., 2002; Wang et al., 2012). GluN3B was once thought to be expressed only in motoneurons (Nishi et al., 2001). However, new evidence reveals that GluN3B is also expressed in the CNS, such as the hippocampus, cerebral cortex, caudoputamen, nucleus accumbens, cerebellum and lumbar sections of the spinal cord (Wee et al., 2008). Compared with conventional NMDARs comprised of GluN1/GluN2 subunits, GluN3-containing NMDARs exhibit several different properties, including lower amplitude currents and lower Ca^2+^ permeability (Low and Wee, 2010; Henson et al., 2010), which suggest that GluN3 subunits play a negative modulation role on NMDAR activity.

Several review articles have summarized the functions of the GluN3A subunit. Specifically, it influences the dendritic spine density, synapse maturation, memory consolidation and cell survival, and it may be involved in certain neuropathologies (Henson et al., 2010; Low and Wee, 2010). The neuroprotective roles of GluN3A subunits have also been proved (Nakanishi et al., 2009; Wang et al, 2012). Cultured neurons prepared using GluN3A-knockout mice display greater sensitivity to damage from NMDA, and cultured neurons expressing transgenic GluN3A display greater resistance to NMDA-mediated neurotoxicity than the wild-type neurons. Similarly, *in vivo*, adult GluN3A transgenic mice subjected to focal cerebral ischemia show less damage than wild-type mice.

The expression of GluN3A subunits shows a temporal profile (Wong et al., 2002; Wang et al., 2012). From postnatal day 7 (P7) to P21, there is an obvious decrease in the protein levels of GluN3A, which remains low into adulthood (Wong et al., 2002). It is clear that the low expression of GluN3A in the physiological condition will hinder its function to execute. Therefore it is worthwhile exploring what happens to GluN3A subunits during brain ischemia and hypoxia. In the present experiment, the influence of brain ischemia and hypoxia on the expression of GluN3A protein is examined using the rat 2VO model and cell OGD models. It is found that, among all the NMDAR subunits, only GluN3A protein expression is raised remarkably in the hippocampus and the prefrontal cortex. GluN1 expression shows little increase, whereas GluN2B expression decreases at first, increases after 3 h, and retunes to the original level at 24 h after ischemia. However, the expression of GluN3B subunit remains stable in the animal and cell models (Figures 2 and 3). The increased expression of GluN3A subunit protein could be detected only 10 min after occlusion, and remains at a high level for at least 24 h. As the expression of GluN3B protein does not show any visible changes during brain ischemia and hypoxia, it is suggested that the GluN3B subunit is probably not involved in the pathological processes evoked by ischemia and hypoxia.

In the present study, it is demonstrated that the expression of GluN3A is inducible during the ischemia and hypoxia process. The quick up-regulated expression of GluN3A makes it credible that GluN3A could play a neuroprotective function. Coincidently, other scientists (Martínez-Turrillas et al., 2012) have also proved that the expression of GluN3A subunits protects vulnerable populations of striatal neurons from excitotoxic damage caused by the mitochondrial neurotoxin 3-NP. They also show that GluN3A-mediated neuroprotection is dose-dependent, which is also consistent with our previous results (Wang et al., 2012).

To evaluate the effects of up-regulated GluN3A subunits in the brain under ischemic and hypoxic conditions, overexpression cell models are made. We compare cell viability of rat hippocampal neurons exposed to OGD with and without GluN3A transfection. The hippocampal neurons overexpressing GluN3A present more pronounced ability of resisting cellular damage than the wild-type neurons in the OGD model. To verify that the protective function is directly related to suppressing NMDARs and reducing neural excitotoxicity, we compared the viability of NG108-15 cells with glutamate insult with and without GluN3A transfection. NG108-15 cells overexpressing GluN3A display a lower injury rate than the wild-type cells exposed to glutamate. Both MTT and FCM assays display the lowest death rate in NG108-15 cells overexpressing GluN3A with pretreatment of MK-801, a non-competitive NMDAR antagonist. The results are consistent with previous reports (Chrysanthy et al., 1999; Pohl et al., 1999) that MK-801 might induce neuroprotection. They also proved that MK-801 could increase neuronal apoptosis in the immature brain, which is consistent with our findings that the apoptotic rate of cells pretreated with MK-801 is higher than those without MK-801 pretreatment in GluN3A overexpressing cells after glutamate insult (Figure 7).

The pathophysiology of brain ischemia is complicated and involves numerous processes and mechanisms. Ca^2+^ overloading has been confirmed to trigger excitotoxicity and result in neuron damage during the brain ischemia process. Because excessive Ca^2+^ influx into neurons is a crucial step for excitotoxicity, we investigate the influence of GluN3A overexpression on the intracellular Ca^2+^concentration. Administration of glutamate and glycine is found to markedly increase intracellular Ca^2+^ levels in wild-type NG108-15 cells. When cells are pretreated with MK-801, the influx of Ca^2+^is totally suppressed, suggesting that the increase in intracellular Ca^2+^ levels is predominantly evoked by activation of NMDARs. The increase in intracellular Ca^2+^ levels also nearly disappears in GluN3A overexpressed NG108-15 cells (Figure 8A). It is suggested that suppression of the increase in intracellular Ca^2+^ levels may be the cause of the neuroprotective mechanism of GluN3A. It is known that GluN1/GluN3 channels are less permeable to Ca^2+^ than the conventional NMDARs. Activation of GluN1/GluN3 receptors evoke far less Ca^2+^-triggered Cl_2_ current than GluN1/GluN2 receptors when Ca^2+^is present in the external solutions (Chatterton et al., 2002). Our results imply that the NMDARs exhibit dramatically decreased Ca^2+^ permeability when excessive GluN3A subunits present in cells. Of course more evidence is needed to demonstrate the existence and increase of NMDARs comprised of GluN1/GluN3 or GluN1/GluN2/GluN3.

Since high levels of hydroxyl radical and NO are involved in ischemia-induced neuron damage, we also evaluated changes of hydroxyl radical and NO concentrations in cells overexpressing GluN3A. Our results reveal that cells overexpressing GluN3A show much lower concentrations of hydroxyl radicals and NO than wild-type cells after glutamate exposure (Figures 8D and 8E). It is suggested that suppression of hydroxyl radicals and NO generation may also be involved in cell protection because the expression of GluN3A is up-regulated during ischemia and hypoxia.

In conclusion, our results demonstrate that brain ischemia and hypoxia could induce rapid up-regulation of the expression of the GluN3A subunit, but not the GluN3B subunit. Overexpression of GluN3A could play a neuroprotective role during the pathophysiological processes of ischemia and hypoxia. Blocking the increase in intracellular Ca^2+^ concentration may underlie the neuroprotective mechanism of GluN3A, and suppression of hydroxyl radicals and NO generation may also be involved in the neuroprotection.
